# Key barriers and enablers associated with uptake and continuation of oral pre-exposure prophylaxis (PrEP) in the public sector in Zimbabwe: Qualitative perspectives of general population clients at high risk for HIV

**DOI:** 10.1371/journal.pone.0227632

**Published:** 2020-01-13

**Authors:** Makaita M. Gombe, Brigid E. Cakouros, Getrude Ncube, Nonhlanhla Zwangobani, Portia Mareke, Alec Mkwamba, Marta R. Prescott, Taurai Bhatasara, Michael Murwira, Alexio Z. Mangwiro, Margaret L. Prust

**Affiliations:** 1 Demand Driven Evaluations for Decisions (3DE), Clinton Health Access Initiative, Harare, Zimbabwe; 2 HIV Prevention, Ministry of Health and Child Care, Harare, Zimbabwe; 3 Technical Directorate, Zimbabwe National Family Planning Council, Harare, Zimbabwe; 4 Applied Analytics Team, Clinton Health Access Initiative, Boston, United States of America; Rutgers School of Public Health, UNITED STATES

## Abstract

**Background:**

Understanding the perspectives and preferences of clients eligible for pre-exposure prophylaxis (PrEP) is essential to designing programs that meet clients’ needs. To date, most PrEP programs in limited-resource settings have been implemented by partner organizations for specific target populations, but the government of Zimbabwe aims to make PrEP available to the broader population at substantial risk in public sector clinics. However, there is limited information on general population perspectives about PrEP in Zimbabwe.

**Methods:**

A qualitative study was conducted to explore clients’ motivation to take or decline PrEP and continue or discontinue PrEP. Through a PrEP pilot in one urban family planning clinic and one rural youth center in Zimbabwe, 150 HIV-negative clients screened as being at high risk of HIV and were offered PrEP between January and June 2018. Sixty semi-structured interviews were conducted with clients who agreed to follow-up (including 5 decliners, all from the rural youth center, and 55 accepters, with 42 from the rural youth center and 13 from the urban family planning clinic). Interviews were conducted after either the first or second PrEP follow-up appointment or after the client declined PrEP. Interviews were audio recorded, de-identified, transcribed, and coded thematically.

**Results:**

PrEP uptake was driven by risk perception for HIV, and in many cases, that risk was introduced by the unsafe behavior or HIV-positive status of a partner. Among sero-discordant couples (SDCs), the desire to safely conceive a child was also cited as a factor in taking PrEP. Clients who opted for PrEP preferred it to other forms of HIV prevention. SDCs reported decreased condom use after PrEP initiation and in some cases were using PrEP while trying to conceive a child. After initiating PrEP, clients had more confidence in their sexual relationships and less stress associated with negotiating condom use. Family and partner support was critical to starting and continuing PrEP, but some clients stopped PrEP or missed appointments due to side effects or logistical challenges such as transportation.

**Conclusions:**

Results of this study can be used to provide operational guidance for national public sector roll-out of PrEP as part of combination HIV prevention in Zimbabwe. Based on feedback and experiences of clients, the training materials for health workers can be refined to ensure that health workers are prepared to counsel clients on the decision to start and/or continue PrEP and answer common client questions. Program advertisements should also be targeted with key messages that speak to client experiences.

**Trial registration:**

Clinical Trial Registry Number: PACTR201710002651160.

## Introduction

Oral pre-exposure prophylaxis (PrEP) with daily tenofovir disoproxil fumarate (TDF) and emtricitabine (FTC) by HIV-negative people can prevent contracting HIV and is a key intervention that can support reducing HIV infections by 90% by 2030. Initially, in 2012, the World Health Organization (WHO) recommended PrEP for special populations such as sero-discordant couples (SDCs), men who have sex with men (MSM), and transgender women who have sex with men [1–4]. However, in 2015, WHO expanded their recommendation to include all population groups at substantial risk of HIV infection as part of a comprehensive package of HIV prevention interventions [5]. The UNAIDS fast track programme identified Zimbabwe as one of the countries that contributes to 89% of new HIV infections globally [6]. These countries are encouraged to take up new innovations such as PrEP in order to meet their 95-95-95 targets by 2030 and reduce the number of new infections. Zimbabwe has a generalized epidemic [7] and would benefit from offering comprehensive HIV prevention and treatment services to the general population.

Other evidence exists on the factors related to uptake and continuation of PrEP in sub-Saharan Africa; however, this evidence comes almost exclusively from participants in clinical trials or other population-targeted programs. PrEP has been described by clients as an HIV prevention tool that allows the user to control their life with minimal cooperation from their partner, especially when condom negotiation is difficult or as additional protection [8]. Perceived risk is suggested to be key for uptake and support for adherence to PrEP by partners and has been highlighted as critical to continued use [9]. In Kenya, Tanzania, and South Africa, adherence was also facilitated by establishing a routine for taking PrEP [10]. However, stigma was also expressed as a barrier to taking PrEP [11].

In Zimbabwe, PrEP was included in the 2016 National Guidelines for Antiretroviral Therapy for the Prevention and Treatment of HIV. In Zimbabwe, an estimated 38,000 new adult HIV infections occurred in 2018, up from 32,000 in 2016. Young women aged 15–24 years account for 9,000 new infections compared to 4,200 young men in the same age group. Women account for 60% of people living with HIV (PLHIV) [12]. Infection rates are higher among sub-populations, such as adolescent girls and young women (AGYW), SDCs, and MSM. PrEP was first introduced in Zimbabwe in private sector demonstration projects for the aforementioned populations. However, the Ministry of Health and Child Care (MoHCC) plans to add PrEP to the HIV prevention package in the public sector based on a client’s risk profile. The risk profile is built from information on sexual practices and history in the six months preceding their appointment using a risk assessment tool (**S1 File**) and follows the eligibility guidelines laid out in the MoHCC’s 2016 National Guidelines for Antiretroviral Therapy for the Prevention and Treatment of HIV [13]. Those at risk are offered PrEP, or a client may request PrEP regardless of the risk assessment outcome. In addition to high-risk groups such as MSM or AGYW, other individuals eligible for PrEP may perceive themselves to be at risk of contracting HIV, possibly due to having multiple sexual partners or stable partners of people with multiple sexual partners. Zimbabwe’s growing epidemic of extra-marital affairs popularly known as “small houses” that have been cited as key drivers of Zimbabwe’s HIV epidemic [14–17] coupled with the country’s socio-economic challenges necessitates a broader HIV prevention and targeting strategy. Accessing such individuals may require different programmatic approaches.

PrEP is a potential user-controlled HIV prevention option with important utility, especially when condom negotiation is difficult [18]. Information about motivations for the general at-risk population in a public sector setting choosing PrEP is limited worldwide and particularly for generalized HIV epidemics like Zimbabwe. The objective of this study was to, therefore, explore the experiences of clients that both accept and decline PrEP offered at a public facility. Specifically, we aimed to understand the factors that motivate clients to accept, decline, continue, or discontinue PrEP. Given the resource limitations of the MoHCC, it is important to understand how to effectively deliver PrEP in the public health system, which may not have the level of resources of well-funded early demonstration projects.

## Methods

A qualitative study was carried out from January to June 2018. This study was approved by the Medical Research Council of Zimbabwe (Protocol MRCZ-A-2234) and the Chesapeake Institutional Review Board (Protocol Pro00023165). The MoHCC offered PrEP in two semi-public Zimbabwe National Family Planning Council (ZNFPC) clinics, a parastatal under the MoHCC mandated to coordinate the provision of family planning services in Zimbabwe. The MoHCC plan for PrEP rollout was also planned to start in these ZNFPC sites before scaling up to other public sector facilities. These facilities were chosen because they were expected to be good environments to reach members of the general population who were seeking services such as cervical cancer screening, VMMC, and family planning services. The two sites were selected because they had the highest HIV testing volumes in their respective settlements. The two PrEP learning sites were selected purposefully to include one urban and one rural site, and PrEP implementation at the learning sites was operated with support from CHAI and the Bill & Melinda Gates Foundation. There were no other sites offering PrEP to the general population near the rural site. In the urban setting, two other partner-funded sites were offering PrEP: one site with outreach specifically targeted towards female sex workers and the other with a more general population focus.

All nurses at these sites were trained on antiretroviral therapy (ART) and TDF-FTC-based PrEP by MoHCC. Clients were eligible for PrEP if they were at least 16 years and had an HIV-negative test result. After HIV testing, clients were screened for PrEP eligibility according to a tool based on government guidelines [13] (**S1 File**), and all clients determined to be eligible for and offered PrEP at one of the two sites were eligible for interviews. Clients were offered PrEP on the same day as eligibility was determined and, if they accepted, were given a one-month supply of PrEP on that day. They were then scheduled for a follow-up visit at one month, where they received a three-month supply. Thereafter, they were scheduled for visits every three months and provided with a three-month supply at each visit. During recruitment, the intervention was not formally advertised; however, clients were informed about PrEP when the nurses gave general health education to all clients during the appointment intake. Nurses would then proceed to conduct client HIV risk assessment during routine consultation. Once a nurse determined a client to be at risk, they would then speak to the client about HIV prevention in general and the possibility of them taking up PrEP. Thereafter, word of mouth from the clients served as the main means of advertising. In the rural site, the Ministry held a meeting with the local chiefs and village heads in order to gain acceptance of the intervention. The village heads proceeded to sensitise their communities on the intervention, and the nurse counsellor at the ZNFPC site would actively recruit clients based on his knowledge of the community around the site.

Interview candidates were categorized into five sub-groups to ensure diversity of perspectives, including people in the following categories (abbreviated with letters for later reference) (1) declined PrEP (D); (2) accepted PrEP but missed their first scheduled appointment (AM); (3) accepted PrEP and attended their first scheduled appointment (AA); (4) accepted PrEP, attended their first follow-up, but did not attend their second follow-up appointment (AAM); (5) accepted PrEP and attended their first and second follow-up appointments (AAA).

**Fig 1:**
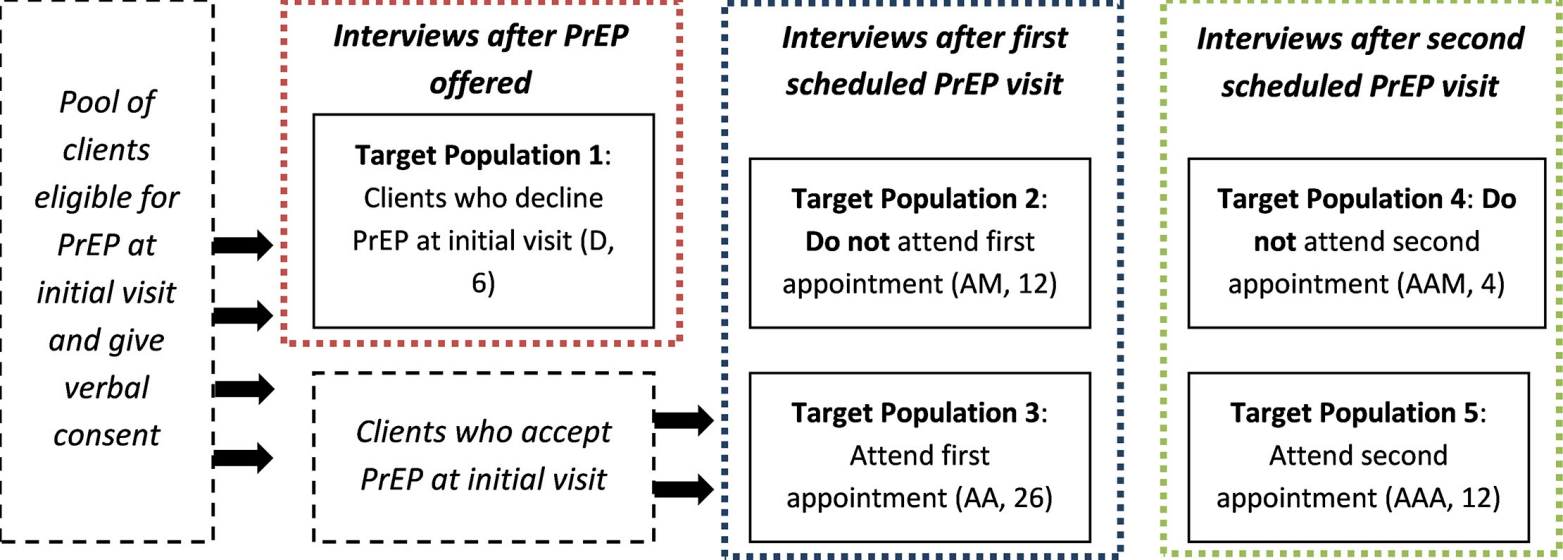
Client interview flow and number of interviews conducted.

**Fig 1** shows the timing of the interviews with each population and the number of interviews conducted with each group.

For clients found to be eligible for and offered PrEP, health care workers (HCWs) described the study and requested their permission to be contacted for an interview. If an individual agreed to be contacted, MMG contacted them by phone. Follow-up phone calls were conducted only for the purpose of recruiting participants and not to encourage retention. Clients that declined PrEP were called approximately one week after they declined, and clients that accepted PrEP were called approximately 7–10 days after either their first or second scheduled appointment regardless of whether they had attended or not. During the initial call, the study was again described. If clients agreed to participate in an interview, an in-person interview was arranged. Written informed consent was obtained from participants at the beginning of the interview.

Interviews were conducted and recorded by Zimbabwean interviewers in English or Shona. Aside from the participant and interviewer, no one else was present. Interviewers were trained qualitative data collectors with no link to the communities. Preferences from clients about interviewer gender were respected. Interviews were conducted in-person at the PrEP site or at the client’s preferred location. The interview guide was based on the Extended Health Belief Model that explores health behaviour constructs, including perceived susceptibility, perceived severity, perceived benefits, perceived barriers, cues to action and self-efficacy [19]. **S2 File** and **S3 File** are the interview guides used for clients who declined or accepted PrEP, respectively. Interviews lasted 15–60 minutes with the average being 45 minutes. Participants were provided with an allowance of US$8 for transport and other costs of participation. Based on a previous VMMC study in Zimbabwe, an $8 allowance was deemed to cover costs without manipulating clients to participate in the study.

Interviews were conducted and analysed on a rolling basis over five months. Recordings were de-identified, transcribed and translated by the interviewer, and the study coordinator (MMG) listened to the audio recordings and checked all the transcriptions to ensure accuracy. Three members of the research team (MMG, MLP, BEC) participated in coding the transcripts, using the constant comparative method to systematically code the transcripts [20, 21]. In the beginning, all members of the coding team independently coded the first three transcripts looking for key themes emerging from study participant responses. Based on this initial reading, the team created a codebook that was then applied to subsequent interviews and adjusted as needed. When all members of the coding team had triple-coded approximately half of the transcripts with any coding discrepancies discussed in the group to reach a consensus, the final codebook was agreed. At this point, the team switched to having two coders code each transcript. At the end of the coding process, the final codebook was applied to all transcripts coded earlier in the process to ensure all interviews followed the same codebook. The coding team provided on-going feedback to interviewers about topics that should be further explored and determined when theoretical saturation was achieved based on review of code reports. Dedoose software was used to organize coded data.

## Results

Three PrEP decliners declined to be interviewed and many more refused to be documented by the nurses as having declined PrEP. The characteristics of the sample and the sub-population they were a part of is shown in **Table 1**. In this paper, each quotation will be identified using the gender, age, location of services (RUR/URB), primary risk factor, and sub-population (D, AM, AA, AAM, AAA).

**Table 1:** Interview participant information^1^.

*Characteristic*	*Number*	*Percent of total*
**Age**		
16–25	12	20%
26–40	36	60%
41 and over	12	20%
**Gender**		
Male	14	23%
Female	46	77%
**Location of services**		
Rural youth center (RUR)	42	70%
Urban family planning clinic (URB)	18	30%
**Sub-population**		
1. Declined PrEP (D)	6	10%
*Interviewed after first scheduled PrEP visit*		
2. Accepted PrEP but missed first visit (AM)	12	20%
3. Accepted PrEP and attended first visit (AA)	26	43%
*Interviewed after second scheduled PrEP visit*		
4. Accepted PrEP, attended first visit, but missed second visit (AAM)	4	7%
5. Accepted PrEP, attended first visit, and attended second visit (AAA)	12	20%
**Primary HIV risk factor**		
Sero-discordant couple	32	53%
Transactional sex or commercial sex work	6	10%
Sexual partner of unknown HIV status	22	37%

1. Note: This table represents the sample size of interviews rather than unique participants.

**Fig 2:**
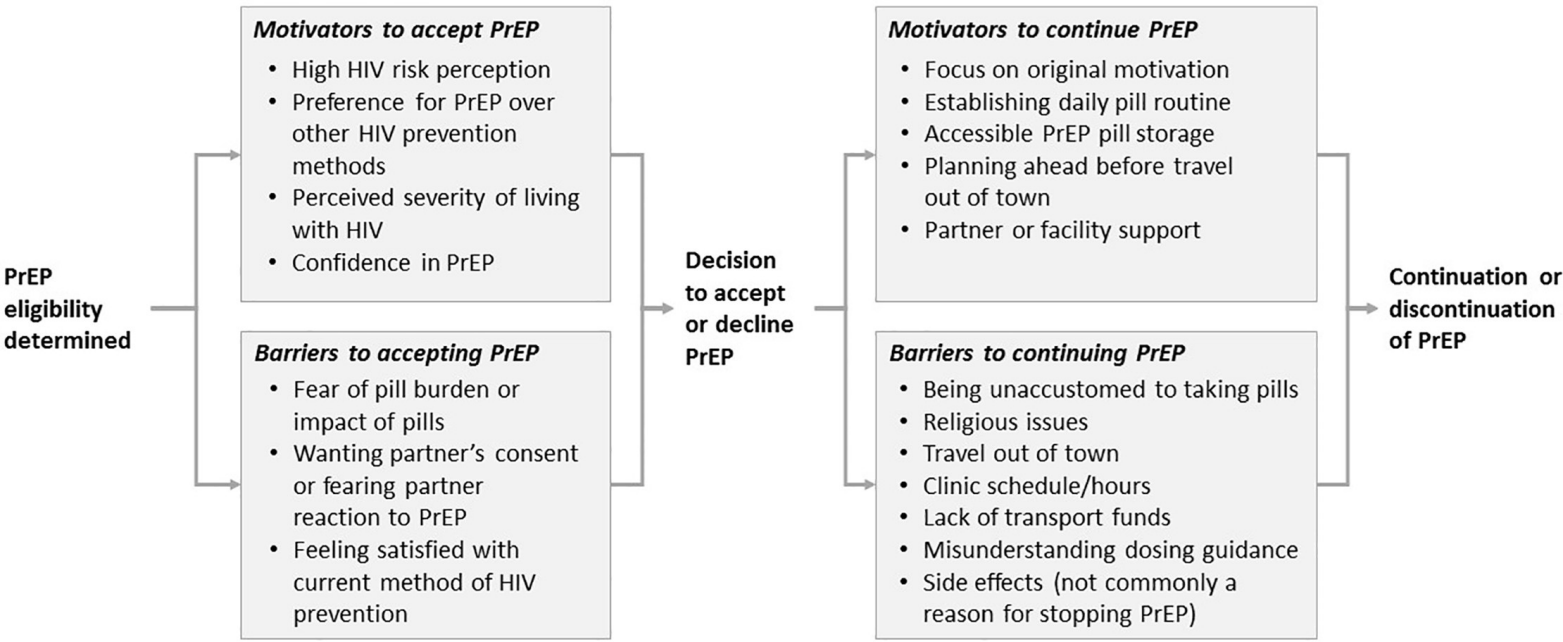
Key factors motivating PrEP uptake and continuation.

This paper presents key themes on factors that motivate clients to accept, decline, continue or discontinue PrEP. The emerging factors are summarized in **Fig 2** and described below.

### Facilitators and barriers for PrEP uptake and continuation

#### High HIV risk perception

Participants who considered themselves to be at high risk of contracting HIV were often interested in PrEP and continued taking it. Most individuals acknowledged being a part of an established risk group, such as sero-discordant couples (SDCs) or commercial sex workers (CSWs), or were concerned their partner may expose them to HIV due their confirmed or suspected extramarital affairs. Almost all men who took up PrEP were in SDCs and the other variations in risk profiles were among women. SDCs indicated a willingness to take PrEP indefinitely in order to live a “normal” sexual life with their HIV-positive partner.

“*My husband and I were quarrelling at home because he was not taking his ART medication*. *Then he would want to be so defensive and harsh and I told him that we cannot stay together if you are not protected I feel uncomfortable so you should use protection and he used protection…*. *My husband defaulted ART the whole of 2017 starting from 2016/2017 and he stopped taking the medication now so he is sick*. *I was offered PrEP and I accepted*. *Now we no longer quarrel”–Female*, *59*, *in SDC (RUR06) (AAA)*

Also, among SDCs, conception without the risk of contracting HIV was also cited as motivation for taking and continuing PrEP.

“*The main reason that made me decide to take PrEP was that I wanted a child*. *[…] all along I tried going to the doctors and that was very expensive*. *I am now nearing a tricky age*. *So when I heard about PrEP I was very happy*.*”–Female*, *37*, *in SDC (URB01) (AA and AAA)*

Many participants not in SDCs also described concern that their partner could expose them to HIV because they observed risk factors in their partners’ behavior issues such as having recurring STI infections, receiving sexual messages on their phones, and refusing to get tested for HIV.

“*I saw a lot of messages from different girls on my husband’s phone*, *I spoke to him about it*, *but I was surprised to be diagnosed with an STI twice*, *I realized I was talking to myself and decided to take PrEP*.*”–Female*, *32*, *partner HIV status unknown (URB06) (AM)*

One participant acknowledged that if she had multiple concurrent partnerships, her partners may also have other sexual partners that could in turn increase her risk for HIV.

“*Sometimes you can get carried away and you go ahead and do it unprotected… If I have two boyfriends*, *how many girlfriends do they have*?*”–Female*, *23*, *partner HIV status unknown (URB09) (AM)*

Finally, those that engage in sex work reported being highly aware of their HIV risk; therefore, they were motivated to get regular HIV testing and to accept PrEP when offered.

“*When I heard about PrEP*, *I came running because the nature of my job*. *[…] I can meet an individual who is HIV-positive in my job*. *God blessed me so far that I am HIV-negative but I knew I was not going to continue to be skillful*, *so I decided to [take PrEP] and protect myself*.*”–Female*, *31*, *CSW (RUR04) (AAM)*

#### Confidence in PrEP as an HIV prevention method

Most participants felt confident that PrEP was effective in preventing HIV, and as a result, they felt empowered that HIV prevention was in their hands.

“*PrEP changed the way I stay with my husband*. *I always thought of divorcing him and going to my parents’ home and work for myself there*, *without any stresses because he would get infected with HIV where he does his promiscuity*. *But from the time that I started taking PrEP I am now a mother who thinks of improving her household*, *without thinking of divorce*.*”–Female*, *28*, *partner HIV status unknown (RUR12) (AA)*

Several female participants mentioned that they are able to use PrEP where condom use presents challenges.

“*I will take PrEP for life because I can no longer be infected by HIV*. *In addition*, *my husband was cruel as he would tear the condoms sometimes and he would pretend as if it had burst*. *I was really happy that I now have backup*.*”–Female*, *32*, *in SDC (RUR33) (AAA)*

PrEP is seen as a way to achieve peace of mind and confidence in being protected, even in cases where a condom breaks or a partner refuses condom use. In fact, using PrEP was even described as being able to allow participants to ask for and enjoy sex more:

“*As a woman*, *[PrEP] can boost your confidence in asking for it [sex] because you know you are protected*.*”—Female*, *37*, *in SDC (URB01) (AAA)*

#### Perceptions of living with HIV

Perceptions and consequences of living with HIV also motivate some people to take PrEP. Generally, participants strongly believed that antiretrovirals (ARVs) work and that HIV-positive people could have “normal healthy lives” if they adhere to medication. They noted that it would be difficult to cope with the infection but a positive mindset was essential to survival. Despite these beliefs, participants were still strongly motivated to prevent HIV. In considering the potential impact of HIV, many participants thought about the impact on their families and their children. Many participants described a fear of loss of economic productivity if they were infected with HIV. They expressed that HIV positive people are too weak to work in their fields or engage in day-to-day activities, and therefore, they and their families would suffer. Female participants expressed a desire to ensure that they remain healthy to care of their children so they wanted to protect themselves from HIV. They expressed concern that if they were no longer there to take care of their children, no one would care for their children as they would.

“*What will really affect me about HIV is that the life that I will be living will hurt me a lot*. *In that now that I will be sick*, *my children pain me a lot because the father will be sick and the mother will be sick*. *That hurts me a lot*. *Who will take care of my children*, *because I will die and that father will die*. *Who will take care of them*, *when we are all gone*? *No one will take care of them and you cannot really on the aunt that they will take care of them because you cannot rely on them and so I should really focus a lot on these pills [PrEP] that will make my life a bit longer as my children grow*, *it is better*.*”–Female*, *28*, *in SDC (RUR01) (AAA)*

“*I liked [PrEP] because we want to take care of our families*. *As women*, *we will not know what we will do with our families [if we are gone]*. *You will be scared of what will happen to your family*. *I did it for my children*. *No one will work for my family if I am gone*.*”–Female*, *33*, *partner of unknown HIV status (RUR03) (AA)*

#### Barriers to PrEP uptake

Some participants were deemed to be eligible, yet chose to decline PrEP and did not attend any follow-up appointments for PrEP. In the PrEP pilot, HCWs did not comprehensively document decliners, so our potential to identify decliners for interviews was limited. However, there were cases where participants who ultimately accepted PrEP described initial hesitation or barriers.

#### Concern for pill burden

A few participants expressed a dislike for pills or concerns about the size of PrEP pills. Several participants indicated that they thought adherence would be difficult.

“*I was just afraid to take pills*. *[…] Growing up*, *I did not take any pills*. *Sometimes if I take even paracetamol the pills will be smelling in my body*. *Whenever I would take the pills*, *I would notice something is happening in my body which will be caused by the pills”–Female*, *42*, *partner HIV status unknown (RUR21) (D)*

#### Needing partner’s consent or fearing partner reaction to PrEP

Whereas some participants appreciated being able to use PrEP privately without consent or knowledge of their partners, other participants said they declined PrEP due to social circumstances such as needing to get permission to take PrEP from their partners. One participant went on to explain that, while she was trying to protect herself from her partner’s risky behavior, he might interpret her taking PrEP as a sign of her own risky behavior.

“*I declined PrEP because my husband would accuse me of having another sexual partner while he is away*. *[…] So I think it is best for me to ask for permission to take PrEP and if he agrees then I will come with him*.*”–Female*, *20*, *partner HIV status unknown (RUR20) (D)*

…. *even if I am faithful but you cannot trust men they can sleep around …*..*I talked to my husband [about taking PrEP] and he told me that he is HIV negative he even asked me that if you want to take the Prep what will be your reasons since we were all tested HIV negative…*. *he did not deny getting tested [on his own]*. *I found it better not quarrel with my partner if he is suggesting other issues rather it will be better to wait for the way forward from him*.*- Female*, *42*, *partner HIV status unknown (RUR21) (D)*

Disclosure of use of PrEP was also cited a consideration for adherence. In some cases, clients received more general emotional support from their partner or family. Simply telling others about PrEP can be liberating or empowering.

“*As for me*, *my brothers*, *mother*, *everyone*, *all my siblings staying here–there is no one who does not know that I am taking PrEP*! *So you find for me it’s easy to keep my bottle in the open and not stashing it in my bag where I can forget it for sure*. *[…] That helps a lot*.*”–Female*, *23*, *two partners*, *HIV status unknown (URB09) (AM)*

While the quotation from the previous participant describes sharing the news of taking PrEP widely, others chose to be more selective. Similar to the decliners, some participants faced challenges in telling their partners about PrEP. They feared being accused of unfaithfulness or promiscuity, therefore opting to discontinue PrEP, especially where conflicts arose.

“*I told my husband that I was taking PrEP*, *and he said I am the one who wants to acquire the HIV that I wanted to prevent*. *And so I realized that it was better for me to just stop because it would create problems*.*”–Female*, *20*, *partner HIV status unknown (RUR34) (AM)*

#### Feeling satisfied with current method of HIV prevention

Another reason for declining PrEP was the preference for condom use. One male participant reported being happy with condoms or other current methods for prevention. Males’ ability to control the use of condoms puts them in a position to decline other HIV prevention methods in comparison to their female counterparts. However, one participant noted that they might consider PrEP in the future.

“*I was told about PrEP and it was ok*, *but I told them that condoms were working well for me and I can stick to them for the meantime*. *But if they were not working well for me*, *I was going to take PrEP*. *[The HCW] said it was ok*., *they were not going to force me to take PrEP*, *because I am the one who knows best on what I need*. *I just want to use condoms only*.*”–Male*, *36*, *in SDC (RUR24) (D)*

### Facilitators and barriers to PrEP adherence and continuation

Participants described the challenges they faced and the approaches they used to help them continue PrEP. This section is organized in terms of facilitators and barriers within the following areas: risk perception, routine and logistics, partner and family interaction, comprehension and counseling, and side effects.

#### High HIV risk perception

From some participants, the continued exposure to perceived HIV risk was a strong motivator to continue PrEP. Most participants in SDCs described feeling that every sexual encounter provides HIV risk exposure, and this motivated them to continue to attend their appointments for PrEP refills.

“*My wife and I agreed to keep on taking PrEP forever unless we get different advice from the health worker*.*”–Male*, *69*, *in SDC (RUR10) (AAA)*

While HIV-negative partners in SDCs described long-term risk perceptions, other participants described how their risk perceptions may change over time, leading to a change in their motivation to continue PrEP. For example, the following participant described how she would stop taking PrEP if her partner agreed to HIV testing:

“*I don’t think I will keep taking PrEP*. *I think that I will be able to convince him [my husband] to go for an HIV test but I am not really sure* … *So even up to now I am scared until I get convinced we can then use condoms*.*”–Female*, *33*, *partner of unknown status (URB12) (AA)*

#### Routine and logistics

Most female participants were already accustomed to the routine of taking family planning pills on a daily basis, so they decided to take all pills at the same time Participants also talked about storing their PrEP and family planning pills together, or in a place where they would easily see it every day.

“*What helps me not forget is that I would like to take good care of my health*. *That is the reason that I placed them where I store my family planning tablets*. *I cannot forget because I want to take care of my family and so I know that if I put them in the same place I will not forget*.*”–Female*, *28*, *CSW (RUR07)*, *(AA)*

Other participants established a routine to help them remember to take their pills daily during the same activity, such as waking up, going to bed, or cooking dinner. Others take the pills at the same time every day, often by setting an alarm on their phones.

“*I take my PrEP around 6pm that is when we will be done with our work in our fields*, *when the sun goes down*. *So when I get into the house before cooking*, *that is when I take my PrEP […]”–Female*, *28*, *partner HIV status unknown (RUR07) (AA)*

However, two participants explained that they had never taken daily pills before and found it difficult to adjust. Similarly, for most, the size of the PrEP pills was a challenge.

“*Just starting to take pills when you have never taken them [is difficult]*. *Plus*, *with how big it was…iiih*! *I would at times take [the pill] and it would take me some minutes feeling that it is still sitting on my throat*.*”–Female*, *29*, *partner HIV status unknown (URB10) (AM)*

Even when clients remembered to or intended to take PrEP daily, some individuals faced logistical challenges. Appointments would be missed due to having gone out of town, such as for a funeral or to buy goods in another country for business. A few were in town, but the hours of the PrEP clinic conflicted with work hours, or they lacked money to pay for transport to the clinic. Transport and logistical challenges were more common in the urban site where some participants were formally employed and also required transport funds to move around.

“*Sometimes I won’t have money*. *Sometimes I will have it*. *Or maybe I have just found enough to go there [facility]*, *but not to come back*.*”–Female*, *31*, *in SDC (URB07) (AM)*

“*It is due to work for the past two weeks [that I missed my appointment]*. *I used to go for day duty and when I am on day duty I work from 7am to 7pm*, *therefore I did not get the chance to go there [to the clinic]*.*”–Female*, *aged 33*, *partner of unknown HIV status (URB11)*, *(AM)*

While travel was a key barrier to returning on time for appointments, some overcame this challenge by planning around their travel. Some participants would travel with enough medication for the duration of their trip or would come to the clinic early if they realized they would run out of pills while traveling. This was observed among both males and females.

“*I came today though my date was tomorrow*. *Because tomorrow I have a journey*, *[I] thought I should come today to minimize any challenges*.*”–Female*, *aged 59*, *in SDC (RUR06) (AAA)*

#### Partner and family interactions

Support or challenges from partners and family members in relation to PrEP was not directly addressed in interview questions, but this issue came up often.

Partners were reported to directly support clients by reminding them to take their pills or about appointments. Indirect support was also provided, such as by providing childcare during appointments. Accounts of these types of support were particularly common within SDCs.

“*[My husband] helps me by asking if I have taken my pills*. *Sometimes I forget*, *so he will be reminding me to take my PrEP*.*”–Female*, *21*, *in SDC (RUR16) (AA)*

In the case where a male participant was taking PrEP because he had multiple wives, including one with HIV, taking PrEP was an effort to protect not only himself but also his other wives. Support for clients to communicate effectively with all parties in such situations is critical. In this case, the man reported the clinic supporting his family by talking directly to the HIV-positive wife at the clinic and sharing written educational materials for other wives.

“*When I first started PrEP it was difficult*, *but I never lost hope*. *[…] It’s very scary to have unprotected sex with an HIV-positive person*. *Even my HIV-positive wife was so afraid asking how we can have sex without using condoms*. *[…] Then I took her to the clinic so that she could understand better from the health workers*, *she accepted the idea of me taking PrEP*. *She was afraid that she will get me infected with HIV because she does not want me to get infected by HIV since I have many wives and I will spread the infection to them*.*”–Male*, *43*, *in SDC (RUR18) (AAM)*

#### Comprehension and counseling

Another challenge with adherence and retention was participants misunderstanding clinical guidance on how often to take PrEP. For instance, an HCW stressed to a client that they should take PrEP for at least seven days (Zimbabwe guidance) before engaging in risky sex to be protected. The participant misinterpreted this as taking PrEP for seven days before expected exposure, with a hiatus until the next expected exposure. Therefore, several months after her original appointment, the participant still had not exhausted her 30-day supply of pills. Another participant describes a similar understanding below of how HIV protection for PrEP works:

“*I am not taking it continuously*. *I take it take a break*, *take it*, *have a break*. *[…] I do not take it so often*. *I took it for the first time just to find out if I had a good relationship with the drug and if I did not have negative side effects occurring*. *I found out that I have a couple of negative effects and then I said*, *I ultimately decided that if I were to take PrEP*, *it has to be seven days before I have sexual encounter which is unprotected with somebody who is not tested*.*”–Male*, *34*, *partner of unknown status (URB02)*, *(AA)*

#### Side effects

Side effects were not reported as a major reason for poor adherence or retention. Many participants reported no side effects. Those who experienced side effects reported them as minor, subsiding after a few days. Reported side effects included headache, dizziness, drowsiness, fatigue, nausea, vomiting, diarrhea, extended or heavy menstrual periods, loss of appetite, and increased appetite. Participants developed coping strategies to deal with side effects, such as eating before taking PrEP pills to avoid nausea or consulting HCWs about them. For example, one participant who experienced nausea found that if she ate a meal before taking her PrEP pills, the symptoms of nausea were not as bad.

“*[Side effects] started from the day I started to take the PrEP pills*. *Sometimes I could even feel a headache*. *I could even feel dizzy and I would also feel sleepy*. *Sometimes I would take paracetamol to manage the headache*, *but as for now the headaches are now better*. *As for now if I take PrEP orally I will feel dizzy*, *so I will take the pills in the evening before I go to bed and I will feel dizzy and sleep*.*”–Female*, *aged 21*, *in SDC (RUR02) (AAA)*

Based on participant responses, having information available about side effects was critical to allowing them to overcome this initial challenge without stopping PrEP. In some cases, participants felt comfortable enough and were able to return to the clinic to report their symptoms to a health worker and get advice:

“*I faced a challenge when I first took my pills*. *I felt weak when I woke up in the morning*. *And I thought I should stop taking [PrEP] but I decided I should not stop without consulting [a health worker] on what was taking place*. *When I consulted they told me that*, *‘I should keep on taking them*. *Maybe they will work well on you*.*’ As for now as I am taking [PrEP]*, *and I no longer feel weak and no more nausea*. *It happened 1 week only*.*”–Female*, *31*, *commercial sex worker (RUR04) (AAM)*

Similarly, another participant went back to the facility to get pamphlets to take home and read for more information about side effects and other issues related to PrEP. For example, the following participant was able to request the information she needed to understand and cope with side effects:

“*I once had a problem of fatigue and dizziness and I decided to come back to ask further about the pill they had given me and how it works because once I took it the next day in the morning when the sun rose I had fatigue and head ache*. *It happened for a few days of which now it has passed*. *[…] I told myself that I will not stop [PrEP] but rather rush to the clinic to ask those who gave me the medication why it happens that way*. *They gave me those pamphlets but at times English is difficult to understand*, *and I asked them to give me Shona ones […] so that I can sit and read comfortably*. *I was given and I read them and understood*.*”–Female*, *28*, *in SDC (RUR01) (AAA)*

There were no differences between the results from rural and populations regarding perceived risks, barriers and facilitators. The only differences observed in these interviews related to how participants found out about PrEP with active demand creation by the nurse in the rural site as opposed to mass media communication or nurse referral from another HIV prevention study in the urban location.

## Discussion

This qualitative study on the introduction of PrEP into the public sector found many themes that will help guide the roll-out of PrEP in the public sector by providing insights into reasons for PrEP initiation and ways to improve PrEP adherence. The effectiveness of PrEP is dependent on its acceptability, accessibility, adoption, and sustainability as part of a comprehensive HIV prevention package. If these components are not there, even the most highly efficacious PrEP medication will have little to no impact in reducing HIV infections [22]. Therefore, HCWs should be trained and familiarized with all themes regarding reasons for initiating, declining, and barriers or facilitators for adherence in order to ensure high-uptake of PrEP within the general population.

Among the participants in this study, who were largely in FSWs or in SDCs or had a partner of unknown status, the perception of risk in these groups of people facilitated their uptake of PrEP, which is consistent with other studies in the United States where PrEP was integrated into a family planning clinic [15]. For all groups, the perceived severity of living with HIV was a key driver of PrEP uptake and the use of PrEP was associated with comfort during sex with an HIV sero-discordant partner. This was also reported in a study among gay bisexual men in HIV sero-discordant male relationships [22]. With regards to SDCs, this work found that women start PrEP as a means to protect themselves from contracting HIV from their partners. In many parts of Africa, the risk of HIV infection is found in marriages where husbands may have acquired the virus through extramarital sex [23–28]. In some cases, the added threat of violence affects women's power and ability to negotiate the conditions of sexual intercourse, especially condom use [26–28]. A woman’s sexuality within the Zimbabwean context is often controlled by her husband or her male partner [28] such that condom negotiation or refusing sex may be difficult. The theme of limited ability to negotiate condom use has emerged in other studies, particularly when considering relationships between AGYW and older men in Africa [29–31]. The quotations from several participants capture the conservative nature of rural Zimbabwe, where staying in a marriage is more respectable than divorce, and therefore, PrEP becomes a means to keep the peace in a marriage. However, a study conducted in Kenya on PrEP acceptability revealed that divorce rates among SDCs were high, highlighting a need to counsel SDCs on how to avoid HIV transmission within the marriage [32]. With regards to male partners in SDCs, the results from this work also show that the men who took up PrEP in this study were in SDCs; however, there was one male decliner preferring condoms to any biomedical HIV prevention method.

The reasons for declining PrEP found in this study included the fear of pill burden, mistrusting of biomedical means of HIV prevention, preferring condoms and discouragement from taking PrEP by family members. These themes are often supported by those found among MSM in the United States [33].

This study showed how critical support, whether from partners or other family and friends, is to PrEP uptake and adherence. Although PrEP may give women the ability to prevent HIV without demanding condom use, many individuals reported difficulty concealing PrEP use from their partners and therefore sought permission before starting PrEP. Accounts from other women in this study described how having support from sexual partners and others improved their adherence. The findings on family and partner support and adherence are supported by evidence from a study among females in Kenya looking at factors affecting PrEP adherence [34]. HCWs should be prepared to counsel clients on talking about PrEP with their partners or family members.

This study was conducted to inform program planning for public sector PrEP rollout by looking at barriers and facilitators to initiation, adherence and retention in an effort to better understand how HCWs can discuss PrEP with clients. The findings from this study provide insight into the types of issues that HCWs and other program staff should be prepared for as they interact with clients around PrEP. For example, sexual relationships can be complex, and HCWs must, therefore, be prepared to counsel clients on their risk level based on a range of situations. Once clients begin taking PrEP, HCWs must ensure that they understand how long they must take PrEP prior to and after possible exposure to ensure maximal protection from PrEP, which may take up to seven days prior to exposure. The counseling needs to stress that daily PrEP is recommended for the majority of persons who cannot predict when they will have sex. However, if their risk changes (e.g., they are not sexually active or have one partner known to be HIV negative), they can discontinue PrEP after at least 28 days after their last exposure. Qualitative studies that were conducted in Kenya and the United States [34–36] also highlight the key roles played by HCWs in counseling clients for PrEP adherence, other prevention methods and community awareness in order to improve PrEP acceptability. Future studies should focus on the perspective of HCWs as they perform counseling and rollout of PrEP to identify ongoing challenges.

The concern that taking PrEP will make clients more promiscuous was not confirmed by participants that were on PrEP. Most participants mentioned that they had not changed their sexual practices or added sexual partners due to the lower perceived risk. Instead, participants continued with the same sexual practices but with less anxiety over contracting HIV after taking PrEP. However, some participants reported decreased condom use after PrEP initiation, and in some cases it was reported that HCWs had recommended this for clients in stable marriages. While other research has shown that stereotypes of promiscuity were a deterrent to taking PrEP in the United States [37], this issue was not raised in the Zimbabwean setting.

### Study Limitations

Few decliners were documented by HCWs. We invited all the decliners for interviews and worked with HCWs to encourage improved documentation of decliners. Participants may have been reluctant to talk about sensitive issues. However, we trained interviewers to help participants feel comfortable in interview settings, matched gender where necessary and held interviews in locations selected by participants. In addition, these participants all self-identified as being motivated to seek PrEP by risk factors related to sexual encounters; this study does not explore access and retention these among other means of risk, such as injection drug use. Information was not available on the HCW perspectives on services and training received as part of this study but should be explored in future studies.

## Conclusions

This study highlights how HIV risk perception, confidence in PrEP, and perceived challenges of living with HIV drove PrEP uptake in this population, while concern about pill burden and side effects and about partner reactions to PrEP, and reliance on other HIV prevention methods led others to choose not to take PrEP. Issues around HIV risk perception, routine or logistics, comprehension and counseling, partner and family support, and side effects were critical in either enabling or prevention clients from continuing PrEP. The introduction of PrEP added a valuable HIV prevention tool to the comprehensive care package in the two study facilities. It has addressed a gap where clients were previously not able to use existing HIV prevention methods for whatever reason. Based on feedback in this study, some of the greatest benefactors of PrEP were women who through this option are empowered to take HIV prevention into their own hands in instances where condom negotiation is difficult or whose sexual partners engage in risky behavior. SDCs were a specific at-risk group who required a backup method for protection or an HIV prevention lifeline, and this study shows that this significant unmet need was addressed through the introduction of PrEP as a prevention option. By understanding the factors that influenced uptake and continuation, future programs can be improved to better serve the target population.

Based on client feedback, we offer recommendations about how to enhance HCW training and service delivery models in order to meet the needs of clients and encourage PrEP uptake for those at risk in the general population. For example, clients may need support on disclosure of taking PrEP and planning appropriately for travel to ensure adherence. Also, going forward, the appropriate timing of use of PrEP needs to be clearly explained to HCWs for counseling purposes to clients. There were a few misconceptions among clients about how to take PrEP, especially if clients had intermittent risk exposure. The use of other prevention methods to complement PrEP was also cited as a point that needed clarity from HCWs, and MoHCC can provide some policy guidance on this issue. At the same time, when considering these recommendations, it is important to take into account the human resources constraints on PrEP programs previously highlighted in the literature [38].

These results have been incorporated into policy documents such as the Zimbabwe National PrEP Implementation Plan [39] and Health Care Worker Training Manual [40], and the HIV Prevention, Treatment, and Care Communication Strategy [41]. These findings may also be relevant in other countries interested in expanding public sector PrEP access.

## Supporting information

S1 File PrEP client form and risk assessment. (DOCX)

S2 File Decliner interview guide. (DOCX)

S3 File Accepter interview guide. (DOCX)

## Abbreviations

3DE: Demand Driven Evaluations for Decisions
AGYW: Adolescent girls and young women
ARVs: Antiretrovirals
AVAC: AIDS Vaccine Advocacy Coalition
CHAI: Clinton Health Access Initiative
DFID: United Kingdom Department for International Development
FSW: Female Sex Worker
FTC: Emtricitabine
HIV: Human Immunodeficiency Virus
MoHCC: Ministry of Health and Child Care
MRCZ: Medical Research Council of Zimbabwe
MSM: Men who have Sex with Men
PEP: Post-Exposure Prophylaxis
PrEP: Pre-Exposure Prophylaxis
SDC: Sero-Discordant Couple
STI: Sexually Transmitted Infection
TDF: Tenofovir Disoproxil Fumarate
VMMC: Voluntary Medical Male Circumcision
WHO: World Health Organization
