# Supplementary material for: Key barriers and enablers associated with uptake and continuation of oral pre-exposure prophylaxis (PrEP) in the public sector in Zimbabwe: Qualitative perspectives of general population clients at high risk for HIV
Source: PLoS One. 2020 Jan 13;15(1):e0227632. doi: 10.1371/journal.pone.0227632 (PMC6957335; doi:10.1371/journal.pone.0227632)
Supplement: S1 File — (DOCX) [file pone.0227632.s001.docx]

# Supporting Information

S1 Additional file: PrEP client form and risk assessment

**SECTION A: CLIENT DETAILS**

| **Name of Health Centre: ____________________________________________ Date of registration: _____/______/______**  **Surname: ___________________________ First name(s): _________________________________________________________**   \| **HTS number** \| **P** \| **P** \| **D** \| **D** \| **S** \| **S** \| **Y** \| **Y** \| **Y** \| **Y** \| **#** \| **#** \| **#** \| **#** \| **#** \|  \| \| --- \| --- \| --- \| --- \| --- \| --- \| --- \| --- \| --- \| --- \| --- \| --- \| --- \| --- \| --- \| --- \| --- \|   **Sex:** 🞏 Male 🞏 Female **DOB: _____/_____/_______ Age:** 🞏 15-19yrs 🞏 20-24yrs 🞏 25-34yrs 🞏 35-49yrs 🞏 ≥50yrs  **Marital Status:** 🞏 Married 🞏 Single 🞏 Widowed 🞏 Divorced  **Address: ______________________________________ Contact phone number: _____________________________**  **______________________________________**  **Confirm that the client’s most recent HIV test was conducted within the last 2 weeks and the result was negative**  🞏 Yes 🞏 No **IF NO, CLIENT IS NOT ELIGIBLE FOR PREP WITHOUT RE-TESTING**  **Date of most recent HIV test:** **_____/_____/_______**  **If known, indicate client profile (tick all that apply):** 🞏 Sex worker 🞏 High risk male 🞏 Prior use of PEP  🞏 Positive STI result in past 6 months 🞏 Has HIV+ partner 🞏 Does not know partner’s HIV status 🞏 Pregnant  🞏 Other potential PrEP risk factors: specify: |
| --- | --- | --- | --- | --- | --- | --- | --- | --- | --- | --- | --- | --- | --- | --- | --- | --- | --- |

**SECTION B: SCREENING FORM FOR PREP (MUST BE CONDUCTED AT EACH CLIENT PREP VISIT)**

| **Questions for Client (*Consider offering PrEP)** |
| --- |
| 1. In the past 6 months: How many people did you have vaginal or anal sex with?   🞏 0(zero) 🞏 1 (one) 🞏 2+ (two or more)*****  (If response is 0 (zero) skip to question 7)   1. In the past 6 months: Did you use a condom every time you had sex?   🞏 Yes 🞏 No* 🞏 Don’t Know*****   1. In the past 6 months: Did you have a sexually transmitted infection?   🞏 Yes***** 🞏 No 🞏 Don’t Know*****   1. Do you have a sexual partner who has HIV? 🞏Yes* 🞏No 🞏Don’t Know*   4a. If ‘Yes’: Has he or she been on therapy for 6 or more months? 🞏 Yes 🞏 No***** 🞏 Don’t Know*****  4b. If ‘Yes: has the therapy suppressed viral load? 🞏 Yes 🞏 No***** 🞏 Don’t Know***** |
| **†Consider offering Post-exposure Prophylaxis** |
| 1. In the past 3 days: Have you had sex without a condom with someone with HIV who is not on treatment?   🞏 Yes**†**  🞏 No 🞏 Don’t Know**†** |
| **‡Consider acute HIV** |
| 1. Have you had a ‘cold’ or ‘flu’ like symptoms such as sore throat, fevers, sweat, swollen glands, mouth ulcers, headache, or rash in the past month? 🞏 Yes**‡** 🞏 No 🞏 Don’t Know**‡** 2. **RISK ASSESSMENT CONCLUSION:**   🞏 Client **is at high HIV risk** based on risk assessment criteria 🞏 Client is **not at high HIV risk** and is not eligible for PrEP at this time  🞏 Acute HIV infection suspected 🞏 Post-exposure Prophylaxis (PEP) offered  🞏 Client requests PrEP but is not at high risk based on risk assessment criteria  Reason for requesting PrEP: |

**SECTION C: CLIENT CLINICAL SCREENING RESULTS**

**Only conduct clinical screening for clients at high risk of infection based on risk assessment or those requesting PrEP**

| **¶DO NOT INITIATE PREP** |
| --- |
| 1. Serum creatinine test result: _______ml/min (conduct every 6 months if available)   🞏 ≥60 ml/min 🞏 <60 ml/min**¶** 🞏 Not done, client **does not have** any known renal impairment  🞏 Not done, client **has** known renal impairment e.g. untreated diabetes, uncontrolled hypertension etc.**¶**   1. Syphilis test result: 🞏 Negative 🞏 Positive 🞏 Not done 2. Hepatitis B test result: 🞏 Negative 🞏 Positive 🞏 Not done 3. Hepatitis C test result: 🞏 Negative 🞏 Positive 🞏 Not done 4. Other STI results if positive: ______________________________________________ 5. Pregnancy test (if applicable): 🞏 Negative 🞏 Positive 🞏 Not done (PrEP is not contra-indicated for pregnancy) 6. If applicable, has the client experienced any condition that could cause vaginal inflammation in the last 3 months (e.g. yeast or vaginal infection)? 🞏 Yes 🞏 No 🞏 Unsure   **CLINICIAN ASSESSMENT:**  🞏 **Client is eligible for oral PrEP**  **Client is not eligible for oral PrEP due to** 🞏 Creatinine clearance fail/renal impairment 🞏 Allergy to any medicine in oral PrEP regimen  🞏 Unwillingness or inability to adhere to daily oral PrEP  🞏 Other (specify:_________________________________________________________________________________________________)  **CLIENT DECISION:**  🞏Initiate PrEP 🞏Decline PrEP  **Reason client declines PrEP initiation (tick all that apply):** 🞏 Doesn’t feel at risk 🞏 Worried about adherence 🞏 Worried about side-effects  🞏 Prefers other HIV prevention strategies 🞏 Wants permission from spouse/parents 🞏 Has heard negative things related to PrEP  🞏 Other (specify:_____________________________________________________________________________________________________) |

**SECTION D: PREP MONITORING**

| **Visit No.** | **Appointment date** | **Actual visit date** | **Visit status** | **Pills dispensed (days)** | **Test result if applicable** | | | **Follow-up status** |  |  |
| --- | --- | --- | --- | --- | --- | --- | --- | --- | --- | --- |
|  |  |  |  |  | **Test date** | **HIV result** | **Creatinine result** |  | **Month of status** | **Clinician initials** |
|  | / / | / / |  |  | / / | +/- |  |  | / |  |
|  | / / | / / |  |  | / / | +/- |  |  | / |  |
|  | / / | / / |  |  | / / | +/- |  |  | / |  |
|  | / / | / / |  |  | / / | +/- |  |  | / |  |
|  | / / | / / |  |  | / / | +/- |  |  | / |  |
|  | / / | / / |  |  | / / | +/- |  |  | / |  |
|  | / / | / / |  |  | / / | +/- |  |  | / |  |
|  | / / | / / |  |  | / / | +/- |  |  | / |  |
|  | / / | / / |  |  | / / | +/- |  |  | / |  |
|  | / / | / / |  |  | / / | +/- |  |  | / |  |
|  | / / | / / |  |  | / / | +/- |  |  | / |  |

| **Visit status: INIT** - Initiation visit **E**-Earlier than appt **OT**-On time **L**-Late <2 weeks **D**-Default <90 days  **RE**-Re-initiated on PrEP (had been >90 days off PrEP)  **Follow-up status :** **Px** - Continue active on PrEP **OO** - Client opted out **WTH**-Clinician decision to withdraw **TO**-Transfer out (specify site)  **TI**-Transfer in (specify site) **SC**-Seroconversion (stop PrEP) **D**-Client died **LTFU** - Lost to follow-up  **FILL OUT PREP TREATMENT NOTES FOR EACH OPT-OUT DISCONTINUATION** |
| --- |

**SECTION E: PREP TREATMENT NOTES**

| **Notes: medical history, hospitalizations, TB history, PrEP side-effects, vaginal inflammation, detailed reason for discontinuation, facilitators and barriers, transfer site, allergies. Indicate clinician name (initials) and date with each new note.** |
| --- |
| **ONLY FOR CLIENTS WHO CHOOSE TO OPT-OUT/DISCONTINUE OR ARE WITHDRAWN BY CLINICIAN**  **(use new notes page each discontinuation)**  **Reasons for opt out/withdrawal (tick all that apply**)  🞏Change in risk profile 🞏Plans to reinitiate in the future 🞏Mild side-effects  🞏Serious side-effects 🞏Creatinine level/renal comorbidities 🞏Partner/parent concerns 🞏 Prefers other HIV prevention strategies  🞏 Other - *specify*: |
