# Supplementary material for: Key barriers and enablers associated with uptake and continuation of oral pre-exposure prophylaxis (PrEP) in the public sector in Zimbabwe: Qualitative perspectives of general population clients at high risk for HIV
Source: PLoS One. 2020 Jan 13;15(1):e0227632. doi: 10.1371/journal.pone.0227632 (PMC6957335; doi:10.1371/journal.pone.0227632)
Supplement: S2 File — (DOCX) [file pone.0227632.s002.docx]

| **Interview Questions** | **Optional Probes** |
| --- | --- |
| **HIV testing:** Why did you decide to get tested for HIV at your last appointment? | - Was there a specific reason for getting tested or is this routine? |
| **PrEP screening**: I understand that a health worker recently offered for you to start taking pre-exposure prophylaxis (PrEP) pills to prevent HIV. Can you tell me what the conversation you had with the health worker was like? | - What kinds of information did the health worker share with you about PrEP? - Did you have any questions that the health worker couldn’t answer? - Did you feel comfortable speaking with the health worker about PrEP? - Was this the first time you had heard about PrEP being offered? What was your understanding of PrEP before this appointment? |
| **PrEP uptake decisions**: What was the main factor that made you decide not to start taking PrEP? | - What were some of the other issues you considered when you decided not to take PrEP? - Is there anything that might make you change your mind about PrEP? |
| **Perceived severity for HIV:** What are your thoughts concerning the seriousness of HIV? What impact could it have on your life if you were to acquire HIV? | - For yourself, family, or career? How easy or difficult would it be to live with the condition? - Can you tell me what you know about how people manage HIV? Do you feel treatment is effective? Why or why not? |
| **HIV risk**: Do you feel that you are currently at risk for HIV? | - Do you take measures to reduce your HIV risk? - Do you feel that taking PrEP could reduce your risk? |
| **PrEP referral**: Would you recommend PrEP to others in your community? | - Why or why not? - Are there some people for whom PrEP would be a better option than others? |
| **Programmatic suggestions**: The government is going to be offering PrEP for free in public health clinics. Do you have any suggestions for how the delivery of PrEP can be improved? | - What kinds of messages should they include in counselling clients to decide if they want PrEP? - Are there any ways that you think health workers can help you to overcome the challenges you talked about today? |
